# A Dimensional Diagnostic Strategy for Depressive Disorders

**DOI:** 10.3390/jcm14030844

**Published:** 2025-01-27

**Authors:** Scott B. Patten

**Affiliations:** Mathison Centre for Mental Health Research & Education, Hotchkiss Brain Institute, University of Calgary, 4th Floor, Cal Wenzel Precision Health Building, 3280 Hospital Drive NW, Calgary, AB T2N4Z6, Canada; patten@ucalgary.ca; Tel.: +403-220-8752

**Keywords:** depression, major depressive disorder, distress, dimensional models, epidemiology, longitudinal data, population data

## Abstract

**Background/Objectives:** Depressive disorders are diagnosed using categorical definitions provided by DSM-5 and ICD-11. However, categorization for diagnostic purposes fails to account for the inherently dimensional nature of depression. Artificial categorization may impede research and obstruct the achievement of optimal treatment outcomes. **Methods:** The current study utilized a Canadian historical dataset called the National Population Health Survey (NPHS) to explore a simple alternative approach that does not depend on categorization. The NPHS collected complete data from 5029 participants through biannual interviews conducted in 1994–2010. Data collection included the K6 Distress Scale as well as the Composite International Diagnostic Interview Short Form for Major Depression. Data from the National Population Health Survey (NPHS) were used to quantify vulnerability to depressive symptoms through longitudinal K6 Distress Scale assessments. Variability of symptoms across this dimension of apparent vulnerability was quantified using ordinal regression, adjusting for age and sex. **Results:** Predicted probabilities from these models were used in simulations to produce a visualization of the epidemiology and to explore clinical implications. **Conclusions:** Consideration of these two dimensional factors (estimated overall level of vulnerability to depression and variability over time) is already a component of clinical assessment and is also accessible to repeated measurement in settings adopting measurement-based care. More formal consideration of these elements may provide a complementary approach to categorical diagnostic assessment and an opportunity for greater personalization of care and improved clinical outcomes. Future studies should validate these findings in diverse clinical settings to ensure their applicability in real-world contexts.

## 1. Introduction

Categorical diagnostic definitions are a longstanding tradition in medicine. Even in the case of clearly dimensional physiological features (for example, blood pressure), categorization provides valuable support for clinical decision-making. For example, when similar diagnostic definitions are used in clinical practice and in randomized controlled trials, evidence from those trials can be more easily translated into clinical practice. Administrative functions, e.g., applications for insurance or disability support, also depend on diagnostic categories. In psychiatry, the most common approach to diagnosis since the publication of DSM-III [1] is the use of diagnostic criteria. However, in the case of depression, there is a downside to diagnostic categorization. This arises because of the broad spectrum of expression of depressive symptoms and their variable nature over time, along with an inevitable loss of information whenever dimensions are reduced to categories. At the mildest end of the spectrum of depression, variability in mood is not pathological, whereas at its other end, it represents a disabling and dangerous health state.

Since depressive symptoms occur across such a broad range of expression, the general approach to creating categories has been to use thresholds. In DSM-5, the diagnosis of a major depressive episode (MDE) is partially based on a threshold applied to symptoms listed in the “A” criterion [2]. The criterion requires certain symptoms to be present (depressed mood, or loss of enjoyment) and a total of five of nine specified symptoms, also specifying that the symptoms be sufficiently severe to affect functioning [2]. ICD-11 adopts a similar, but not identical, approach [3].

The dimensionality of depression, however, is not limited to symptom severity. The persistence of symptoms is also an important dimension, and DSM-5 requires that symptoms persist “most of the day, nearly every day” for at least 2 weeks [2]. Here, a temporal threshold (14 days) is applied. Deciding when an episode of depression has ended requires an additional threshold that differs from that used for diagnosis. DSM-5 specifies that a remission means that there are “no significant symptoms” which is a lower threshold than five of nine symptoms most of the day nearly every day, as required to identify the onset of an episode. Also, in order to represent a remission, this state of “no significant symptoms” should persist for 2 months, resulting in the application of a fourth threshold, this time another temporal one. ICD-11 does not have the latter temporal requirement for remission [3], such that its definition only applies three thresholds rather than four.

The application of thresholds to dimensional variables always leads to a loss of information [4]. For illustration, one may consider depression-screening scales such as the PHQ-9 [5,6,7]. This is a nine-item scale, each item having four item-response categories scored 0 to 3, leading to a minimum score of 0 and a maximum score of 27. When the standard cut-point of 10 is used as an interpretive threshold, the resulting dichotomous scoring equates a score of 0 with a score of 9 and a score of 10 with a score of 27. This represents a loss of information since such differences in scores often reflect important clinical differences. When one considers that the DSM-5 criteria require the application of four distinct thresholds to four distinct dimensional characteristics, it is unsurprising that, in certain scenarios, the diagnostic categorization arising may provide a misleading representation of clinical realities. For example, very different patterns of symptoms over time may appear identical through the application of those criteria, and similar patterns may appear starkly different [8].

In addition to a loss of information, the use of thresholds may introduce artifactual complexity into the epidemiology of depression. For example, high levels of symptoms at baseline (e.g., what might be called subclinical “episodes”) are predictors of subsequent incident episodes [9,10,11,12,13], and similarly “residual symptoms” are considered risk factors for relapse [14,15]. In both instances, the patterns may be artifactual in the sense that people falling closer to a threshold are more likely to cross it, even in the case of minor variability over time, such that a change in diagnostic status does not necessarily represent an altered health state. A person close to the threshold may enter or leave a diagnostic category after a small or insignificant change in their symptoms, whereas those further away from a threshold may not have a changed diagnostic status even if there is a large change. This is an example of the loss of information that occurs with categorization. Despite the differences in terminology, residual, prodromal, and subthreshold symptoms have similarities [16,17], and it is not clear that different labelled categories are required for them.

In terms of epidemiology, categorization of dimensions may lead to an unnecessary increase in complexity. Many such complexities characterize the epidemiological literature about major depression. For example, the rate of recovery from a major depressive episode diminishes as the duration of that episode lengthens [18,19,20]. This is an apparent example of a time-varying recovery rate, but may merely reflect the categorization of a dimension of vulnerability to symptoms. Similarly, the observation that depression with earlier onset tends to be more severe [21] may merely represent more severe disorders resulting in earlier (with “earlier” here conceived in terms of age rather than time) crossing of diagnostic thresholds. The duration of time in “remission” predicts a lower chance of relapse [22] and the number of past episodes is a predictor of future episodes [23,24]. These are all apparent complexities that may arise simply as artefacts of the application of thresholds.

In mathematical models of depression epidemiology, exponential distributions have not been found to adequately reflect incidence or remission, suggesting that there is no rate of incidence or remission. Rather, the time, event, and age-varying rates have often been depicted using more complex distributions such as the lognormal and Weibull distributions [18,25] or Markov tunnels [26]. For example, the Weibull distribution has a shape parameter that allows a rate to change over time. However, if the time-dependent changes are merely artefacts of categorization, then the use of such strategies in modelling may diminish their parsimony and utility.

DSV-5 and ICD-11 both allow for mild, moderate, and severe versions of depression in their diagnostic definitions, adopting something of a dimensional perspective. However, these attributes are assigned after an episode is diagnosed. This is an “after-the-fact” subcategorization that does not address the problems listed above.

A dimensional perspective on depression might simply focus on symptom severity, thereby adopting a purely descriptive stance. Instead, the dimensional perspective offered in this manuscript emphasizes a dimension of vulnerability (or diathesis) to depression. The goal is not to understand the factors underpinning this diathesis, such as genetics, epigenetics, and childhood adversities [27,28] or affective temperaments, which have been viewed as representations of the genetically determined substrate of mood disorders [29]. Rather, the diathesis is viewed as an observable and assessable feature in clinical practice, a feature of a patient’s history. The goal of this paper is to explore the potential utility of such a dimension in providing an alternative perspective on diagnosis. This aligns with a dimensional framework’s focus on persistent vulnerability rather than episodic symptoms.

The goal of this paper is to explore an extremely simple framework for the conceptualization of depression as a dimension using an approach that maintains clinical utility and may provide a framework complementary to traditional diagnoses. The framework was organized around statistical parameters estimated from historical data from a study called the National Population Health Survey (NPHS) collected between 1994 and 2010 [30,31,32]. The framework is presented in the forms of (1) a set of quantitative results, (2) a visual animation based on a simple simulation model, and (3) a statistical analysis of output from the model. These results are presented as a framework for dimensional diagnosis rather than as a theory of psychopathology or a fully validated and calibrated model (the data source would be insufficient for these purposes). However, the empirical foundations of this simple model in epidemiological data allow a more meaningful set of clinical and epidemiological implications to be identified from it. These should be regarded as hypotheses for future work.

## 2. Materials and Methods

### 2.1. Data Source

The NPHS was a national panel study conducted in Canada between 1994 and 2010. The study was conducted by Statistics Canada, Canada’s national statistical agency, with longitudinal data made available to researchers through a national network of Regional Data Centres [33]. Detailed information about the survey is available from various prior publications [31,32] and online sources [30,33].

In brief, the goal of the NPHS was to collect information on the health of the population, including relevant sociodemographic data [31]. The target population consisted of residents of Canada’s 10 provinces, but excluded residents of reserves and Crown Lands, residents of institutions, full-time members of the military living in Canadian Forces Bases, and a few remote areas in the provinces of Quebec and Ontario. These exclusions represent only 2–3% of the national population, but mean that NPHS estimates should not be generalized to these populations.

The NPHS used a stratified two-stage sampling design that was based on two different sampling frames, one for Quebec and one for the other 9 provinces. The stages of sampling identified households in the first stage and then selected one individual from each selected household in the second phase. In the first “cycle” of data collection in 1994/1995, approximately 75% of the interviews were conducted in person and the rest by telephone. Starting in the next cycle, the vast majority (about 95%) of the interviews were conducted by phone. In each case, computer-assisted interviewing was used [30].

The NPHS sample initially consisted of 17,276 persons, including some participants under the age of 12, whose data collection occurred by proxy (through their parents), and where the intention was that these participants would later participate in full interviews as they reached the age of 12 during the longitudinal follow-up. These participants were excluded from the current analysis, as were those who died during follow-up, were lost to follow-up, were institutionalized during follow-up, or where proxy interviews were required during follow-up (e.g., due to development of dementia), as the mental health measures were not administered by proxy. Individual-item non-response was very low in the NPHS (generally less than 1%). Participants with missing items were excluded from the current analysis. Imputation was not used. The current analysis is based on 5029 participants who were over the age of 12 and who provided complete data at each of nine follow-up intervals. Restriction to participants having complete data was considered acceptable given the goals of the study.

The complex sampling strategy of the NPHS leads to a requirement for use of “master” sampling weights and a set of 500 replicate bootstrap weights. All of the epidemiological estimates from the NPHS presented in this paper have used this approach [30].

### 2.2. Measures

The analysis used four variables from the survey dataset. Sex and age were recorded at the baseline interview in 1994/1995. Also, the Kessler-6 (K6) scale ratings obtained at each cycle were used in the analysis. The K6 was not selected because it was considered the best measure of depression symptoms (some of its items refer to anxiety), but it was the best available measure in the NPHS and is a widely used and well-validated scale. The K6 is a six-item general distress scale covering symptoms of depression and anxiety in the preceding month. The items take the following form: “During the past month, that is, from [date one month ago] to yesterday, about how often did you feel so sad that nothing could cheer you up?” “During the past month, that is, from [date one month ago] to yesterday, about how often did you feel nervous?” For each of the six items, the response categories were “All of the time”, “Most of the time”, “Some of the time”, “A little of the time”, and “None of the time”. The items were scored 0–4, allowing for a minimum score of 0 and a maximum score of 24. The K6 was developed for use in population surveys and is widely used in population health studies [34,35,36]. It has good internal consistency and a single-factor structure [37], consistent with the inter-relationship of depression and anxiety symptoms [38].

The NPHS also included a brief diagnostic instrument for major depression called the Composite International Diagnostic Interview—Short Form for Major Depression, abbreviated in this paper as CIDI-SFMD [39,40]. This is a brief, branched interview that asks about whether specific symptoms (approximately reflecting the “A” criterion for major depressive episode) occurred during the same 2-week period during the preceding year. The CIDI-SFMD leads to a predictive probability estimate. The presence of five of the nine symptoms leads to a 90% predictive probability of a past-year major depressive episode [39], providing some evidence of face and construct validity. The CIDI-SFMD was developed for use with the DSM-IIIR diagnostic criteria; however, it operationalizes the symptom-based component of the major depressive syndrome, which has not substantially changed in DSM-5 [2]. The major change between DSM-IV and DSM-5 was the removal of a bereavement exclusion criterion, which was much discussed during the development of DSM-5 [41,42,43], but is not incorporated into the CIDI-SFMD, an approach that is actually more consistent with the DSM-5 than DSM-IIIR approach. Generally, the CIDI-SFMD has produced estimates consistent with those from its parent instrument, the full Composite International Diagnostic Interview [44].

### 2.3. Approach to Modelling

The study required a dimensional construct representing the person-specific experience of depression and anxiety, as a measure of vulnerability to these symptoms. For this purpose, the within-person mean K6 rating was estimated for this purpose. By calculating the within-person level of distress over time, based on the 9 assessments in 1994–2010, it was hoped that the estimate would represent the average vulnerability of each participant to these symptoms, averaging out the within-person variability in symptoms due to associated life events and circumstances as well as less explicable and more random variation over time. Averages were rounded in order to quantify this dimension in integer form: ranging from 0 to 18. For example, scores > 0 and <1.5 were rounded to 1, and the remaining scores were rounded to integers (e.g., average scores ≥ 1.5 but <2.5 were rounded to 2). Due to data release requirements, the scores > 12 needed to be coalesced into a category of 12+ in some parts of the analysis, but all participants were included in the fitting of the ordinal regression models. This resulted in the identification of a dimensional index characterizing differing levels of vulnerability. This index is referenced as a “diathesis” in the text of this paper, a widely used term for vulnerability to an illness.

The CIDI-SFMD was used to produce counts of the number of episodes during follow-up as a function of the K6-defined diathesis, in order to help confirm that the patterns seen in the K6 ratings are relevant to the occurrence of disorders. For this purpose, the K6-defined diathesis levels were compared to the number of MDE episodes to assess the strength of association between these variables.

The proportion of the population falling within each level of diathesis was estimated using an ordinal logistic regression model that included sex (modelled as a binary characteristic) and age (modelled as a continuous variable). In the simulations, the predicted proportions from the ordinal logistic regression were calculated and used to simulate the distribution of diatheses within the population.

Next, within each of the 12 diathesis levels, a second set of ordinal logistic regression models were fit in order to model the predicted values of specific distress levels within each stratum over time. These predicted values were then used to simulate individual K6 scores for a series of observations over time for each simulated participant in the simulation model. Note that there is no concept of episodicity in this approach. Each simulated monthly K6 rating is treated as an observation from a probability distribution according to the ordinal regression models, depending only on age, sex, and the dimension of vulnerability.

The simulation used NetLogo [45]. The model assigned values for sex (*p* male/female = 0.5); a value for age and sex was simulated using probabilities derived from national sources (published estimates from Statistics Canada [46]). The model was used to produce a visual animation and to generate data. Both versions are included as Supplementary File S1 to this manuscript. The visual animation is based on simulation for *n* = 1000 entities. The version of the model that stores output data was used to simulate data for *n* = 200,000 entities. The data were analyzed using Stata [47].

As the simulation approach described above uses no concept of episodes, the data were analyzed through the lens of an episodic condition in order to evaluate the extent to which the complex features described above emerge as artefacts of categorization; see below. Standard thresholds were applied to the simulated data both for incidence (13) and remission (6). As the model uses one-month intervals, consistent with the time period covered by the K6, no specific duration threshold (e.g., 14 days) was applied in order to identify an episode; a monthly K6 score ≥ 13 was sufficient. Remission was defined in the model as having two months with simulated K6 scores less than the remission threshold (K6 < 6). The simulations were run for 192 months in order to be roughly consistent with the duration of the NPHS and, for the same reason, simulated K6 ratings were recorded at 24-month intervals. Four specific hypotheses were explored in the statistical analysis of the simulated data:

(1) Is simulated episode duration consistent with declining remission rates over the course of an episode? The recorded duration of the last full episode in the simulated data was used to produce a description of episode duration.
(2) Does the number of past episodes predict the risk of future ones? To assess this, the probability of being in an episode at the end of the 16-year (192-month) simulation period was stratified by the number of simulated episodes preceding it.
(3) Does time since remission predict recurrence? To assess this, the frequency of episodes at the end of the simulation was stratified by time since the last episode among the simulated entities having an episode.
(4) Do residual symptoms predict recurrence? To assess this, the symptoms at the time of remission from the last full episode were compared between those with or without an episode at the end of the simulation.

## 3. Results

Even though the sample used in the current analysis was restricted to participants with complete follow-up, the weighted demographics of the sample continued to resemble those of the general population. The weighted proportion of women was 53.4% (95% CI: 52.3–54.4). The mean age of the sample at baseline was 36 years. The mean K6 score at baseline was 2.78 (95% CI 2.68–2.7).

**Figure 1:**
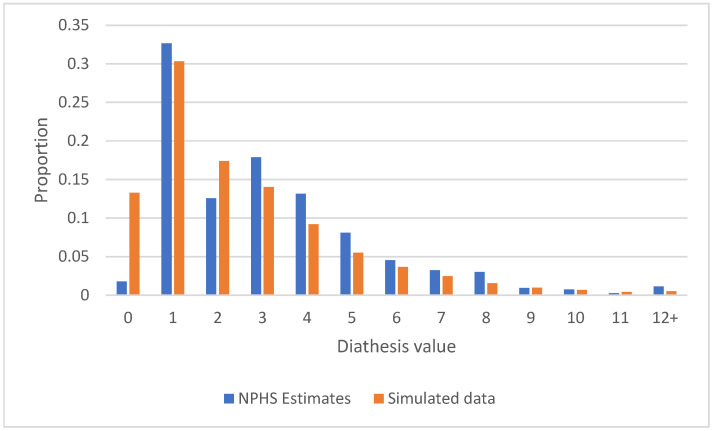
Proportion of the population falling into each diathesis level, NPHS 1994–2010. Simulated values from the simulation analysis are also presented (orange bars).

The estimated proportion of the population falling into each diathesis level is presented in Figure 1, blue bars. The pattern is that most of the population have low levels of diathesis (average K6 scores of <3) and a diminishing proportion have ever-higher levels.

As an example, an ordinal regression model depicting variability in scores at level one of the diathesis is presented in Table 1.

**Figure 2:**
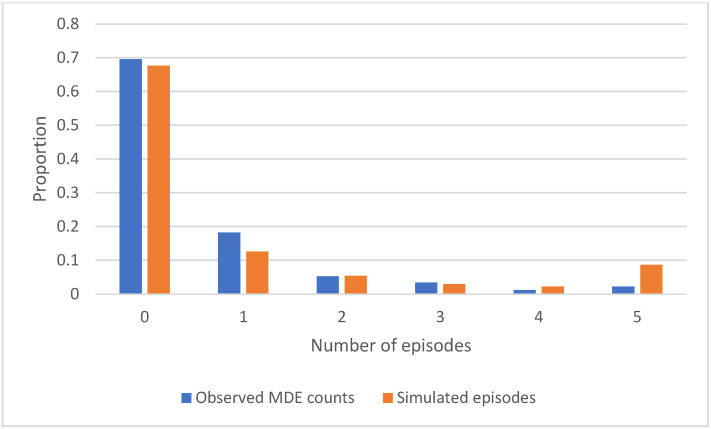
Observed and simulated episodes over 16 years with biannual sampling.

Counts of MDE over 16 years of follow-up, according to the CIDI-SFMD, were strongly related to the diathesis variable in an ordinal regression model adjusting for age and sex: Coeff = 0.5606716, bootstrap SE = 0.0277077, z = 20.24, and *p* < 0.001. In the simulation, episodes of simulated K6 ≥ 13 had a similar frequency distribution to the number of CIDI-SFMD-defined past-year episodes; see Figure 2. The same pattern was previously reported from the first eight cycles of the NPHS [48]. This helps to confirm that the K6 thresholds identify a similar pattern to the CIDI-SFMD data.

**Figure 3:**
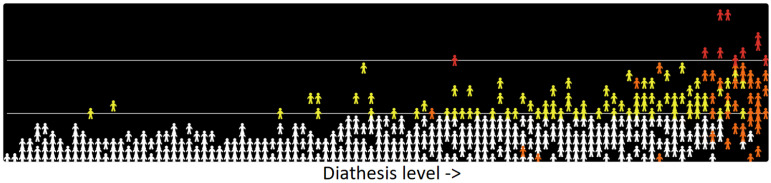
Layout of the visual animation. Level of symptoms is simulated on the vertical axis, with diathesis level on the horizontal axis. Thresholds used in the simulation are depicted by horizontal white lines.

The visual animation is presented as an html file, which can be opened in a web browser (“model for visual animation.html”), by following this link [49]. A brief screen capture of the running model is available in this link [50]. In order to see the visualization in html format, the button labelled “clear” should be clicked first; next, clicking “simulate diathesis” will assign a diathesis value to each of the simulated entities (there is a slider where the sample size can be changed) and sort them with the lowest values to the left and highest values to the right. This step illustrates that the diathesis level is low for most of the population and is increasingly high for an increasingly small proportion of the population. The cut-points for the diagnostic and remission thresholds can be adjusted using the sliders labelled “DxCutpoint” and “RemissionCutpoint” and can be added to the animation by clicking the buttons below those sliders. To start the simulation, the “go” button needs to be clicked. The simulated entities, which retain their positions with those having the lowest diathesis on the left to highest diathesis on the right, move up and down as predicted by the ordinal regression models described above. Recall that the movement represents random predicted value from the diathesis level with no concept of episodes. When above the diagnosis cut-point, they are coloured red; if below the remission cut-point- they are white- and between the two cut-points (between the diagnostic threshold and the remission cut-point), they are yellow, representing “subthreshold” or “prodromal” symptoms. The orange entities are those that have not been in remission long enough to return to white (two consecutive months below the remission threshold). A screen capture of an animation is presented in Figure 3.

The animation illustrates the advantages of thinking in dimensional terms. Even though clinical episodes occur at low diathesis levels, these are likely to be brief and unlikely to recur; the majority of the clinical pathology occurs at higher levels of diagnosis. Longer episodes occur with increasing frequency at higher diathesis levels. At the highest level of diathesis, chronicity emerges.

After running the “model with recorders”, also included as a Supplementary File S1, the four hypotheses stated above were evaluated statistically. Simulated data and the Stata “do” file used to produce these estimates are also provided in the Supplementary Materials.

The odds ratio for sex was consistently in the range of 1.5 to 1.7 at various time points in the simulation. That for age, when included as a continuous variable in a logistic regression model, was consistently less than 1.0, reflecting the predicted decline in prevalence with age.

**Figure 4:**
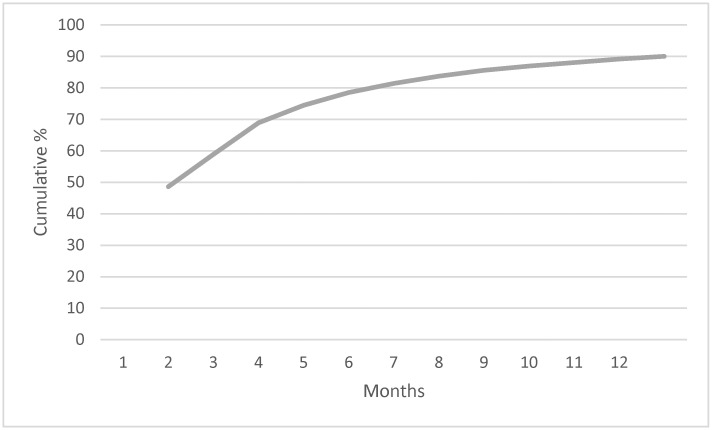
Cumulative frequency of remission, by month.

In examining the pattern of persistence of episodes over time (Hypothesis 1), the pattern was the expected one. In Figure 4, the slope of the cumulative remission curve is the rate of remission, which diminishes, as expected, as episodes become longer in duration.

Hypothesis 2 was also confirmed. Among simulated entities with only one past episode, the incidence of a new episode present at the end of the simulation was 3.5%, which increased progressively with the number of past episodes. Among those with two past episodes, the incidence was 8.6%; for those with three past episodes, it was 15.7%; and among those with four past episodes, it was 24.8%. Among those having at least one episode, those having one within the past 5 years had an OR for recurrence of 3.4 relative to those simulated entities with 5+ years of remission (hypothesis 3). The importance of “residual symptoms” was also confirmed (hypothesis 4). Those with distress levels of 4 or 5 at the time of remission from their last episode had a relative odds of recurrence by the end of the simulation interval of OR of 8.1 (compared to those with symptoms 0–3 at the end of their previous episode).

Speculatively, the effects of treatments (e.g., antidepressant medications) may act by reducing the level of diathesis. This idea applies especially to long-term treatments, since, in the dimensional model, concepts such as resolution of episodes are not relevant. To explore this idea, the simulation model was modified to assign a treatment to 50% of simulated entities. The treatment reduced the simulated diathesis by a specified percentage. In the simulations described below, treatment reduced the diathesis by 35%. Simulations (*n* = 100,000) were run to look at the effects of a treatment on symptoms at specific time points. In the total population, the standardized mean difference for treatment was 0.30 at the 48-month follow-up time point (and was almost identical at all other time points). Among entities with mild symptoms at the preceding (24-month) time point, the SMD was 0.43, whereas among those with scores of 13+ at the preceding time point, the SMD was 0.71.

## 4. Discussion

The main finding of this analysis is that a simple dimensional representation of depression explains key aspects of the epidemiology without resorting to the somewhat arbitrary threshold-based categorizations that have become the most common approach to the diagnosis of depression. Notably, these thresholds have been considered “somewhat arbitrary” due to limited validation data supporting them [51]. This study provides a different perspective on dimensionality than the current diagnostic classifications, which are “after-the-fact” classifications of episode severity, as seen in DSM-5 [2] and ICD-11 [3]. The approach also differs from dimensional strategies based only on symptom severity or on combinations of symptom clusters [52], and from approaches that define a “spectrum” of disorders based on a set of ordered subtypes [17]. The perspective presented here relates more closely to Klein’s proposal for a two-dimensional approach, incorporating symptom severity and chronicity [53]. Shankman and Klein earlier explored the value of adding dimensional features (severity and an indicator of course derived from a principal component analysis) to the DSM categories of major depression and dysthymia, finding that their addition in a set of hierarchical regression models did not incrementally improve the prediction of a set of validation parameters, such as recovery or family history [54].

The simple dimensional model explains many of the complexities of the categorical approach, suggesting that many of the complexities of the epidemiological literature are better regarded as artefacts of a particular approach to categorization. Implications for clinical practice arise because the concept of a diathesis, as conceived here, is accessible to history taking in a clinical interview. This would involve inquiries about the extent of time spent with various levels of burden of depressive symptoms; see also [8]. Rather than focusing on which symptoms were present in the past two weeks, as prioritized by the DSM-5 and ICD-11 definitions, clinicians should focus more on the overall vulnerability of their patients to depression as evidenced by the longitudinal pattern. As depressive symptoms are often responsive to life events, inquiries about experiences of depression at different points in time and in different circumstances will allow patients to be placed, at least qualitatively, onto a dimension of vulnerability. The goal of treatment would then be to reduce the level of vulnerability or diathesis, leading to a change in the long-term pattern of experience of symptoms of depression and anxiety.

In depression therapeutics, measurement-based care is an important and effective strategy [55]. If patient-reported outcomes are routinely being collected in a particular setting, then the concept of diathesis becomes more quantitatively accessible. This quantification may be as simple as repeated application of a measurement scale and calculation of an average. As such, measures each represent an observation from within a population of observations all deriving from a specific person (the within-person concept used in this study).

The unplanned analysis, simulating the effects of treatment, in this study is consistent with the existing literature, showing that antidepressant treatments are stronger, and perhaps only worthwhile, in those with moderate to severe depression, as opposed to mild depression [56]. However, a possible artefact is again present since a relative effect of treatment (reduction in diathesis by 35% in the simulations presented) predictably translates into a larger absolute effect size at higher levels of vulnerability.

An important implication of these results is a challenge against recommendations that full remission should always be a target of treatment. Of course, full remission is the best outcome, but for those with a strong diathesis, it may not be feasible. Diminished vulnerability over time, along with a reduced symptom burden over time, is then seen as a more suitable metric of outcome than achievement of remission from a supposed episode. Clinical success, through a dimensional lens, would manifest as less person-time with problematic symptoms, rather than entry into the state of remission at a point in time. Some authors have previously used similar metrics. Another concern about aggressive pursuit of remission as a goal of treatment is that guidelines often recommend progression through different levels of treatment, proceeding with second- and third-line treatments if remission is not achieved [15]. Second- and third-line treatments are often less evidence-based and more subject to adverse events (e.g., combinations of medications) than first-line treatments, such that the pursuit of remission might lead patients away from effective treatments (dimensionally defined, those that reduce their long-term vulnerability) towards less effective and less safe ones.

Another implication is that a dimensional viewpoint may help to guard against clinical mistakes that can arise due to an excessively categorical view. Often, patients receive effective treatments, which markedly diminish their burden of depressive symptoms over time, but when a spike in symptoms occurs, the assumption is made that the treatment has “stopped working” and a change is made to a new medication. The new medication may or may not be as well tolerated or as effective as the previous one. The results presented here may indicate that the long-term pattern of symptoms should be the target of change, not a temporary (and potentially infrequent) change.

Similarly, in clinical trials of depression treatment, the proportions responding or achieving remission are usually the primary outcome measures. These results suggest that a different design, with multiple assessments before and after initiation of treatment, would provide a better metric of efficacy than an assessment at a point in time.

This paper did not seek to formally validate or calibrate the simulation model against statistical estimates of MDE from the NPHS. For, example, while the pattern of episode counts depicted in Figure 2 resembles that of the NPHS, the two types of episodes are not equivalent. In general, it should be emphasized that the primary purpose of the simulation was conceptual and illustrative, rather than for decision support or prediction.

This study’s limitations include the use of a distress scale for quantifying symptoms, rather than a depression scale. Also, the study was conducted in Canada, which has seasonal [57,58] and geographical [59] variability in depression prevalence, likely making it different from some other countries. The patterns described here may not generalize to other countries. Furthermore, the NPHS data are now dated. It should be noted that the data collection targeted the household population and thereby excludes 2–3% of the national population, including those who are homeless or reside in institutions, full-time members of the Armed Forces, and residents of Indigenous reserves. As the study included only complete responders, selection bias may have been introduced through mechanisms such as attrition, institutionalization, and mortality. While the study used simulation, the model was not calibrated against other sources of population data. The model is a stark simplification of a complex scenario. The goal of the simulation was to illustrate the dimensional perspective, not to guide policy or support clinical decisions.

## 5. Conclusions

In clinical settings, diagnostic assessment is primarily categorical, but assessment and monitoring can benefit from dimensional measurement as well. This manuscript reports a concept of dimensionality, that of vulnerability or diatheses, which is accessible to clinicians through history taking. Consideration of this dimension may enhance clinical judgements in ways that “after-the-fact” attributions (mild, moderate, severe) or symptom scale ratings cannot. This dimensional conception deserves to be evaluated in future research as a component of diagnostic assessment to determine its clinical utility.

## Figures and Tables

**Table 1:** Ordinal regression model for level 1 of diathesis (see Figure 1).

	Observed	Bootstrap		
	Coefficient	Std. Err.	z	*p* > |z|
Female	0.6020257	0.072615	8.29	0
Age	−0.0334561	0.002705	−12.37	0
/cut1	−4.649223	0.130308		
/cut2	−1.1666	0.085626		
/cut3	−0.6045533	0.085363		
/cut4	0.1861806	0.085282		
/cut5	0.8832061	0.089607		
/cut6	1.467017	0.099937		
/cut7	1.93001	0.108008		
/cut8	2.403018	0.123056		
/cut9	3.133108	0.156643		
/cut10	3.508108	0.180597		
/cut11	3.937813	0.232022		
/cut12	4.145967	0.264448		

## Data Availability

Data analyzed in the course of this project are confidential and require analysis in the secure setting of a Regional Data Centre. There is a process for accessing the data within an RDC: https://www.statcan.gc.ca/en/microdata/data-centres/access, accessed on 6 January 2025.

## Supplementary Materials

The following supporting information can be downloaded at https://www.mdpi.com/article/10.3390/jcm14030844/s1, File S1: (1) the NetLogo model used in the visual animation, (2) the NetLogo model used to collect data for analysis of the simulation model, (3) the NetLogo model used in the treatment simulations, (4) the data file from the statistical analysis in Stata (dta) format and comma-separated values, and (6) the Stata “do file” used to produce the statistical estimates presented in the manuscript and (7) an html version of the NetLogo model, which allows the animation to be viewed in a web browser.
