# Supplementary material for: A Dimensional Diagnostic Strategy for Depressive Disorders
Source: J Clin Med. 2025 Jan 27;14(3):844. doi: 10.3390/jcm14030844 (PMC11818090; doi:10.3390/jcm14030844)
Supplement: Supplementary file 1 [file jcm-14-00844-s001.zip › model for visual animation.html]

### Error

NetLogo Web has encountered a problem.

It looks like you're using NetLogo Web in standalone mode.
  
If the above error is being caused by an unimplemented primitive, we recommend a quick visit to
NetLogoWeb.org
to see if the primitive has been implemented in the most up-to-date version.

Dismiss
